# Identification and Application of a Novel Immune-Related lncRNA Signature on the Prognosis and Immunotherapy for Lung Adenocarcinoma

**DOI:** 10.3390/diagnostics12112891

**Published:** 2022-11-21

**Authors:** Zhimin Zeng, Yuxia Liang, Jia Shi, Lisha Xiao, Lu Tang, Yubiao Guo, Fengjia Chen, Gengpeng Lin

**Affiliations:** 1 Division of Pulmonary and Critical Care Medicine, The First Affiliated Hospital of Sun Yat-sen University, Zhongshan Second Road No. 58, Guangzhou 510080, China; 2 Institute of Pulmonary Diseases, Sun Yat-sen University, Guangzhou 510275, China

**Keywords:** immune-related lncRNA, ddPCR, prognosis, immunotherapy, lung adenocarcinoma

## Abstract

Background: Long non-coding RNA (lncRNA) participates in the immune regulation of lung cancer. However, limited studies showed the potential roles of immune-related lncRNAs (IRLs) in predicting survival and immunotherapy response of lung adenocarcinoma (LUAD). Methods: Based on The Cancer Genome Atlas (TCGA) and ImmLnc databases, IRLs were identified through weighted gene coexpression network analysis (WGCNA), Cox regression, and Lasso regression analyses. The predictive ability was validated by Kaplan–Meier (KM) and receiver operating characteristic (ROC) curves in the internal dataset, external dataset, and clinical study. The immunophenoscore (IPS)-PD1/PD-L1 blocker and IPS-CTLA4 blocker data of LUAD were obtained in TCIA to predict the response to immune checkpoint inhibitors (ICIs). The expression levels of immune checkpoint molecules and markers for hyperprogressive disease were analyzed. Results: A six-IRL signature was identified, and patients were stratified into high- and low-risk groups. The low-risk had improved survival outcome (*p* = 0.006 in the training dataset, *p* = 0.010 in the testing dataset, *p* < 0.001 in the entire dataset), a stronger response to ICI (*p* < 0.001 in response to anti-PD-1/PD-L1, *p* < 0.001 in response to anti-CTLA4), and higher expression levels of immune checkpoint molecules (*p* < 0.001 in PD-1, *p* < 0.001 in PD-L1, *p* < 0.001 in CTLA4) but expressed more biomarkers of hyperprogression in immunotherapy (*p* = 0.002 in MDM2, *p* < 0.001 in MDM4). Conclusion: The six-IRL signature exhibits a promising prediction value of clinical prognosis and ICI efficacy in LUAD. Patients with low risk might gain benefits from ICI, although some have a risk of hyperprogressive disease.

## 1. Introduction

Non-small cell lung cancer (NSCLC) remains the second-most commonly diagnosed cancer (with an estimated prevalence of 11.4%) and the leading cause of cancer death (with an estimated prevalence of 18%) in the global population, but incidence declines and survival increases in the United States of America. The tobacco epidemic, ambient air pollution, the diagnosis period, and late-stage treatments likely contribute to the remarkable difference [1]. Lung adenocarcinoma (LUAD), as one of the common and severe lung cancers, accounts for approximately 50% of NSCLC [2] and has an average 5-year survival rate of 21% given to the missed diagnosis in an early stage and advanced cancer refractory to traditional treatments [1]. A large proportion of patients still cannot benefit from current conventional chemotherapy and targeted treatments because of the resistance, which leads to a relatively high recurrence rate in LUAD [3].

Cancer immunologic and immune therapeutic advances seem promising for gaining a survival benefit for LUAD. Increasing studies focused on tumor microenvironments reported that infiltrating immune cells and modulating immune pathways have prominent effects on the progression and aggressiveness of LUAD [4]. Besides, immune checkpoint inhibitors (ICIs) directed against programmed cell death-1 (PD-1) and its ligand (PD-L1) have revolutionized the treatment of advanced LUAD without targetable mutations [5]. Unfortunately, the overall response to ICI is modestly low, and a paradoxical acceleration of tumor growth, defined as “hyperprogressive disease (HPD),” happens in a subset of patients with NSCLC treated with ICI [6]. Thus, the molecular signature relevant to tumor immunology is needed to be recognized as prognostic biomarkers to optimize personalized medicine and improve long-term survival.

Along with advances in immunology, researchers studied the critical regulatory ability of long non-coding RNA (lncRNA) in different phases of cancer immunity, such as antigen release and presentation, immune activation, immune cell migration, infiltration, and killing of tumor cells [7]. Besides, the immune signature can be a conspicuous marker to evaluate the overall survival (OS) in patients with LUAD [8]. However, few studies have comprehensively considered prognostic immune-related lncRNAs (IRLs) and their roles in predicting the efficacy of ICI treatment.

In this study, according to the IRL-based risk model, the subtypes of LUAD were identified to evaluate prognosis, immune cell infiltration, therapeutic benefit, and HPD during immune checkpoint blockade via integrative bioinformatics.

## 2. Materials and Methods

### 2.1. Data Download

Expression, phenotype, and survival data were downloaded from The Cancer Genome Atlas (TCGA (RRID:SCR_003193)) cohort of the UCSC Xena database (https://xenabrowser.net/ accessed on 16 September 2020), in which 513 LUAD samples were obtained as an entire dataset after removing 13 samples with the missing phenotype (Table S1). Then, gene symbol names were retrieved from the human gtf file in the Ensembl database (http://www.ensembl.org/info/data/ftp/index.html accessed on 23 April 2020). A total of 3547 LUAD-associated IRLs were acquired from Lnc_Immunecell_Sig and Lnc_Pathways_Sig files in the immLnc database (http://bio-bigdata.hrbmu.edu.cn/ImmLnc/ accessed on 28 March 2021) [9]. The RNA sequencing data and relevant clinical characteristics of GSE120622 of patients with LUAD were downloaded from the Gene Expression Omnibus (GEO) (https://www.ncbi.nlm.nih.gov/geo/ accessed on 4 July 2022) [10]. Besides, the version 22 reference genome data, immunophenoscore (IPS) data [11], and target mRNAs of IRLs were obtained from The Gencode database(https://www.gencodegenes.org/ accessed on 27 April 2020), The Cancer Immunome Atlas database(TCIA, https://tcia.at/home accessed on 10 May 2021) [11], and starbase3.0 (http://starbase.sysu.edu.cn/ accessed on 6 April 2020) database.

### 2.2. Patients’ Samples

To verify the risk score calculated from the TCGA database, we collected 40 samples of lung tissue punctured by thoracoscope and relevant clinical information as a retrospective case-control study in the First Affiliated Hospital of Sun Yat-sen University between October 2020 and June 2021. In accordance with the International Association for the Study of Lung Cancer tumor–node–metastasis classification [12], 30 and 10 cases were diagnosed with LUAD and nonlung cancer, respectively. The clinical characteristics of patients with LUAD are shown in Table S1. This study was approved and supervised by the Research Ethics Committee of the First Affiliated Hospital of Sun Yat-sen University (No. (2022)049).

### 2.3. Weighted Gene Coexpression Network Analysis (WGCNA)

The WGCNA (RRID:SCR_003302) R package (version 1.69) was used to analyze the co-expression network of IRLs [13]. Specifically, the screening criterion was R-square > 0.85, and the soft thresholding power of four was selected. By using the power of four and a merged module threshold < 0.25, highly correlated clusters were merged into similar modules. Modules were generated, and the hierarchical clustering dendrogram was plotted.

### 2.4. Efficacy Analysis of Risk Score

All samples were randomly divided into TCGA training (360 samples) and testing (153 samples) datasets in accordance with the ratio of 7:3. Combining survival data, univariate Cox regression was performed on the training dataset by using R package survival (version 3.2-7) and R package survminer (version 0.4.8), and a *p* value < 0.05 was set for screening significantly differentially expressed genes [14]. Afterward, the Lasso regression was used to further narrow differentially expressed genes via the R package glmnet (version 4.0-2), in which the minimal lambda was obtained by a cross-validation procedure and then used to fit the Lasso model [15]. According to ${\mathrm{RScore}}_{i}=\sum_{j=1}^{n}\exp_{ji}\times \beta_{j}$ (exp represents gene expression; β represents coefficients of genes identified by Lasso regression; and *i* and *j* represent each sample and each gene), we calculated the risk score of each sample and divided all patients with LUAD into high- and low-risk groups by using the median risk score [16]. Combined with survival data, the Kaplan–Meier (KM) curve was plotted between high- and low-risk groups with a *p* value < 0.05. The receiver operating characteristic (ROC) curves and area under the ROC curves (AUC) were drawn and calculated to estimate the 5-year survival probability [17].

### 2.5. Analysis of Stability and Independence

To validate the stability of the risk score, all patients were stratified into different subgroups on the basis of age (<60 vs. ≥60 years), gender (male vs. female), and TNM stage (stage I + II vs. stage III + IV). The survival status between high- and low-risk groups in KM curves was determined (*p* value < 0.05 defined as significant). We incorporated several parameters, including age, gender, M stage, N stage, T stage, and risk score, into univariate and multivariate Cox regression analyses.

### 2.6. Construction of a Nomogram

By using the R package rms (version 6.1-0) and survival (version 3.2-7), the prognostic nomogram was plotted with clinical parameters after establishing the Cox proportional hazards model and calculating survival probability [18]. The ROC curve was used to validate and predict the nomogram.

### 2.7. Assessment of Immune Microenvironment

The gene expression *signature* matrix of LM22 was obtained (CIBERSORT, RRID:SCR_016955) to estimate the proportions of 22 types of infiltrating immune cells, and expression levels were calculated in all samples by using the R package CIBERSORT (version 1.03) [19]. The R package estimate (version 1.0.13) was used to calculate the stromal score, immune score, ESTIMATE score, and tumor purity of all samples to compare the difference between high- and low-risk groups. Besides, univariate and multivariate Cox regression analyses were used to analyze the clinical traits and risk scores of LUAD.

### 2.8. Evaluation of Response to ICIs

IPS-PD1/PD-L1 blocker and IPS-CTLA4 blocker data on LUAD from TCGA were obtained in TCIA for predicting patients’ responses to ICI in high- and low-risk groups. In addition, the expression levels of immune checkpoint molecules and markers for HPD were compared in these two groups.

### 2.9. Gene Set Enrichment Analysis

The Hallmark pathway enrichment analysis was performed using gene expression profiling data from different groups with LUAD by a GESA software (version 3.0) from Sangerbox (http://vip.sangerbox.com/home.html accessed on 27 July 2021) [20] In the GSEA runs, gene-set sizes were set to between 5 and 5000 and parameters were set to 1000. A *p* value < 0.05 and an FDR < 0.25 were considered statistically significant.

### 2.10. Droplet Digital PCR

The Digital PCR MicroDrop-100 and Reagents (Kitforevergen) were used for droplet digital PCR (ddPCR) experiments. Primers are shown in Table S3. The cycle parameters used were as follows: 95 °C for 10 min and 95 °C for 30 s; 40 cycles of 60 °C for 30 s, and 72 °C for 1 min, and 72 °C for 30 s and final heating at 16 °C. The QuantaSoft software was used to analyze the number of copies of each sample.

### 2.11. Statistical Analysis

The R software (version 4.0.5) or GraphPad Prism (version 7.0) was used for statistical computing and graphics. For continuous variables, a *t*-test was used if data were normally distributed, *whereas the Mann–Whitney test* or Wilcoxon was performed for data that did not follow a normal distribution. For categorical variables, the chi-square test was performed. The univariate Cox regression analysis was used to examine potential risk factors, and the multivariate Cox regression analysis was further carried out for covariates whose *p* value < 0.05 in the univariate analysis. The KM curve was plotted to analyze the differences in survival by the log-rank test. Relationships between modules and traits were analyzed by the Pearson correlation. A *p* value < 0.05 represented statistical significance in all analyses.

## 3. Results

### 3.1. Identification of Prognostic IRLs

**Figure 1:**
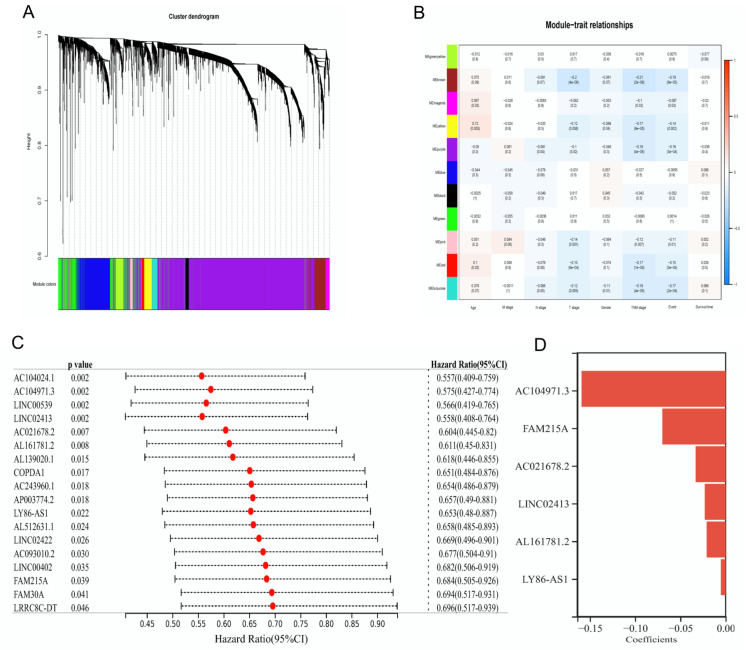
Six identified immune-related lncRNAs (IRLs) after weighted gene coexpression network analysis (WGCNA) and regression analyses. (**A**) Hierarchical clustering tree on IRL’s coexpression network. (**B**) A heatmap of modules and clinical traits, including age, M stage, N stage, T stage, gender, TNM stage, clinical event, and survival time. The numbers in each box and parenthesis represented correlation coefficient and *p* value. (**C**) Eighteen prognosis-related IRLs screened by univariate Cox regression. (**D**) Coefficients of six prognostic IRLs identified by the Lasso model.

Integrative bioinformatics was conducted to explore the prognosis, immune cell infiltration, therapeutic benefit, and HPD of LUAD. To obtain relevant IRLs from core modules in LUAD, we acquired 513 TCGA–LUAD samples with complete clinical information (Table S1) and 3547 LUAD-associated IRLs. The WGCNA was then conducted to categorize the expression pattern of IRLs in TCGA–LUAD samples into 11 similar modules via the hierarchical clustering dendrogram at the appropriate soft threshold power of four (Figure 1A). Module–trait relationships were analyzed by correlating the 11 modules with clinical characteristics. We found that the brown co-expression network, including 174 IRLs, had negative associations with TNM stage and clinical events (Figure 1B).

To further narrow significant prognosis-related IRLs, we identified 18 candidate IRLs (*p* < 0.05) by investigating the association between the expression levels of 174 IRLs and survival information in the TCGA training dataset by using univariate Cox regression (Figure 1C). Afterward, six prognostic IRLs were identified as the key immune signature by Lasso regression with a minimal λ value (Figure 1D) and clustered in heatmaps (Figure S1). Based on the coefficients of the six IRLs, an optimized prognostic model for LUAD was constructed by calculating the risk score as follows: risk score = AC104971.3 × (−0.1592) + FAM215A × (−0.0697) + AC021678.2 × (−0.0330) + LINC02413 × (−0.0228) + AL161781.2 × (−0.0207) + LY86-AS1 × (−0.0051).

### 3.2. Predictive Capability and Sensitivity of the Risk Score Model

**Figure 2:**
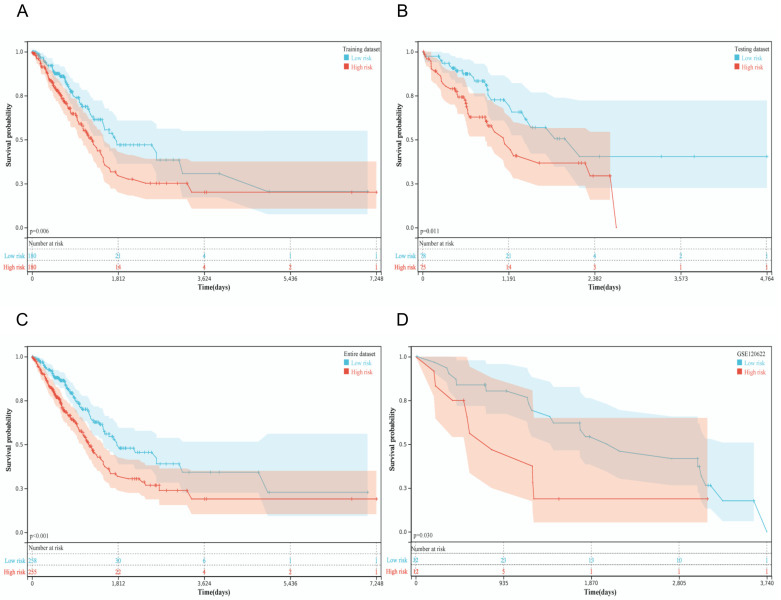
Survival prognosis of the risk score model. Kaplan–Meier (KM) survival curves of patients with LUAD in high- and low-risk groups from (**A**) TCGA training dataset, (**B**) TCGA testing dataset, (**C**) TCGA entire dataset, and (**D**) GSE120622.

Aiming to assess the predictive value of the constructed prognostic model with six IRLs, patients were divided into low-risk and high-risk groups according to the median risk score of −0.46. The KM curve was plotted to compare the survival time in the TCGA training dataset, revealing that the median survival time of the high-risk group was shorter than that of the low-risk group (*p* = 0.006, Figure 2A). Afterward, KM curves were also drawn in the TCGA testing dataset (*p* = 0.010), TCGA entire dataset (*p* < 0.001), and GSE120622 (*p* = 0.040) to validate the predictive ability of the prognostic model (Figure 2B–D). These KM curves showed that patients with LUAD in the high-risk group had a worse prognosis than those in the low-risk group. The ROC curves of the 5-year OS indicated that the risk score was essential in predicting prognosis in patients with LUAD (TCGA training dataset: AUC = 0.64; TCGA testing dataset: AUC = 0.64; TCGA entire dataset: AUC = 0.64; GSE120622: AUC = 0.62; Figure S2), which indicated a predictive ability based on the risk score.

Furthermore, we verified the risk score by directly detecting the absolute quantification of these six IRLs via ddPCR in LUAD samples and controls from a case-control study in south China. Consistent with the results from the TCGA and GEO datasets, patients with LUAD and high-risk scores exhibited poor outcomes in our clinical study (Table 1). Except for the predictive power of the IRL signature, we wondered whether these six IRLs expressed differently between LUAD and normal controls. Results showed that AC104971.3 (*p* < 0.01), AC021678.2 (*p* < 0.01), LINC02413 (*p* < 0.05), AL161781.2 (*p* < 0.05), and LY86-AS1 (*p* < 0.01) were significantly downregulated in clinical LUAD tumor tissues, and that FAM215A expression was not significantly different but slightly increased in tumor samples (Figure S3). Besides, patients with LUAD from the TCGA dataset, GEO dataset, and clinical study were divided into high- and low-risk groups to avoid baseline bias. No difference was observed between the two groups in gender and age (Table S2).

**Figure 3:**
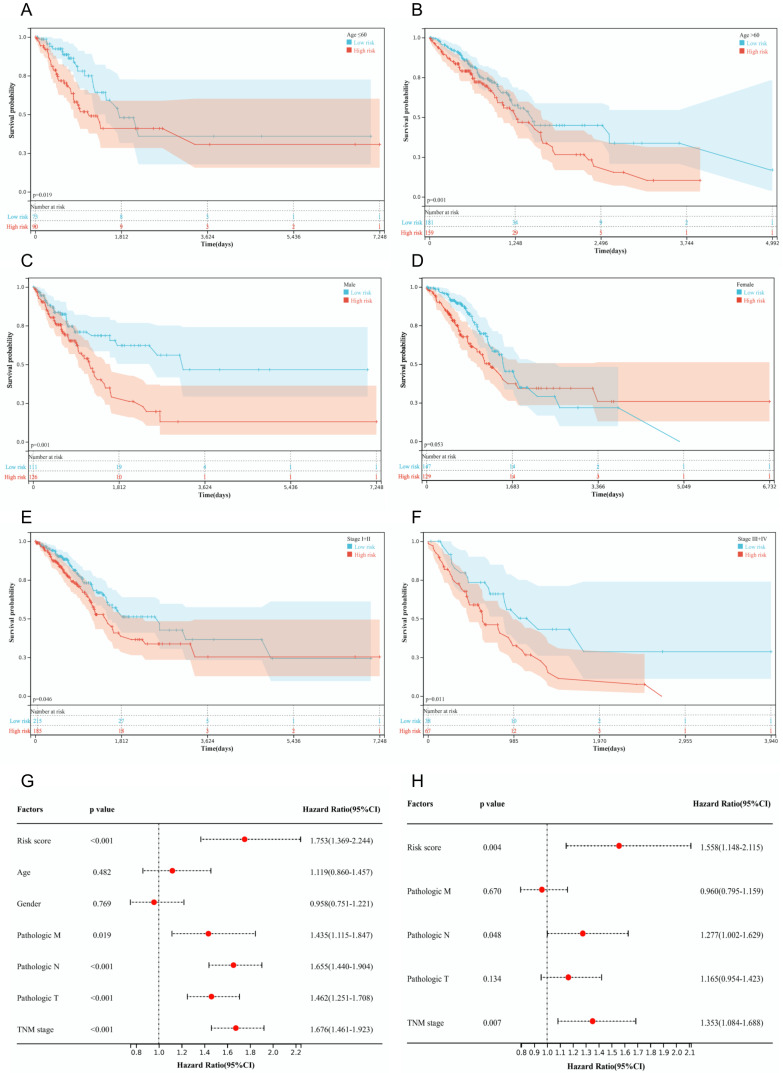
Stability and independence of the prognostic risk model. Survival times of patients among different (**A**,**B**) age, (**C**,**D**) gender, and (**E**,**F**) TNM stage subgroups. (**G**) Univariate and (**H**) multivariate Cox regression analyses on risk score, age, gender, M, N, T, and TNM stages.

Additionally, to prove the stability and independence of the risk score based on IRLs, we plotted KM curves on patients with LUAD under different subgroups divided by baseline characteristics and performed univariate and multivariate Cox regression analysis on the risk score and various clinicopathological characteristics. Results showed that patients in the low-risk group had a high chance of survival at a different age (*p* = 0.019 in age ≤ 60 years, *p* = 0.001 in age > 60 years), TNM stages (*p* = 0.046 in stage I + II, *p* = 0.011 in stage III + IV), and male patients (*p* = 0.001 in male patients; Figure 3A–F). Univariate and multivariate Cox regression analyses demonstrated that the risk score (*p* = 0.004) and TNM stage (*p* = 0.007) had a remarkable predictive capability when considering potential risk factors (Figure 3G,H). Hence, the established risk score had an independent and reliable prognostic performance in predicting patients with LUAD.

### 3.3. Construction of an IRL Signature-Based Nomogram

**Figure 4:**
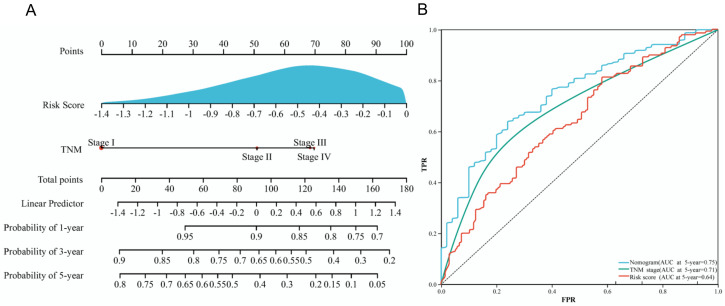
Nomogram integrating risk score and clinical features. (**A**) Nomogram for predicting 1-, 3-, and 5-year overall survival rates of patients with LUAD. (**B**) ROC curves of the risk score, TNM stage, and nomogram. AUC, areas under the ROC curve. FPR, false positive rate. TPR, true positive rate.

A nomogram was established to visualize the above independent factors, including risk score and TNM stage (Figure 4A), in which the ROC curve showed that the AUC of the 5-year survival probability in the nomogram was 0.75 (Figure 4B). This finding suggested that the complex nomogram integrating IRLs and clinical characteristics could be effective in predicting the survival status of LUAD. Besides, we compared several IRL prognostic models and relevant nomograms of LUAD in a published paper [21,22,23,24]. Similarly, the nomogram AUC values of these four TCGA entire datasets were more than 0.70, but the nomogram AUC of GSE120622 was between 0.58 and 0.75 (Figure S4).

### 3.4. Immune Landscape and Efficacy of ICI

**Figure 5:**
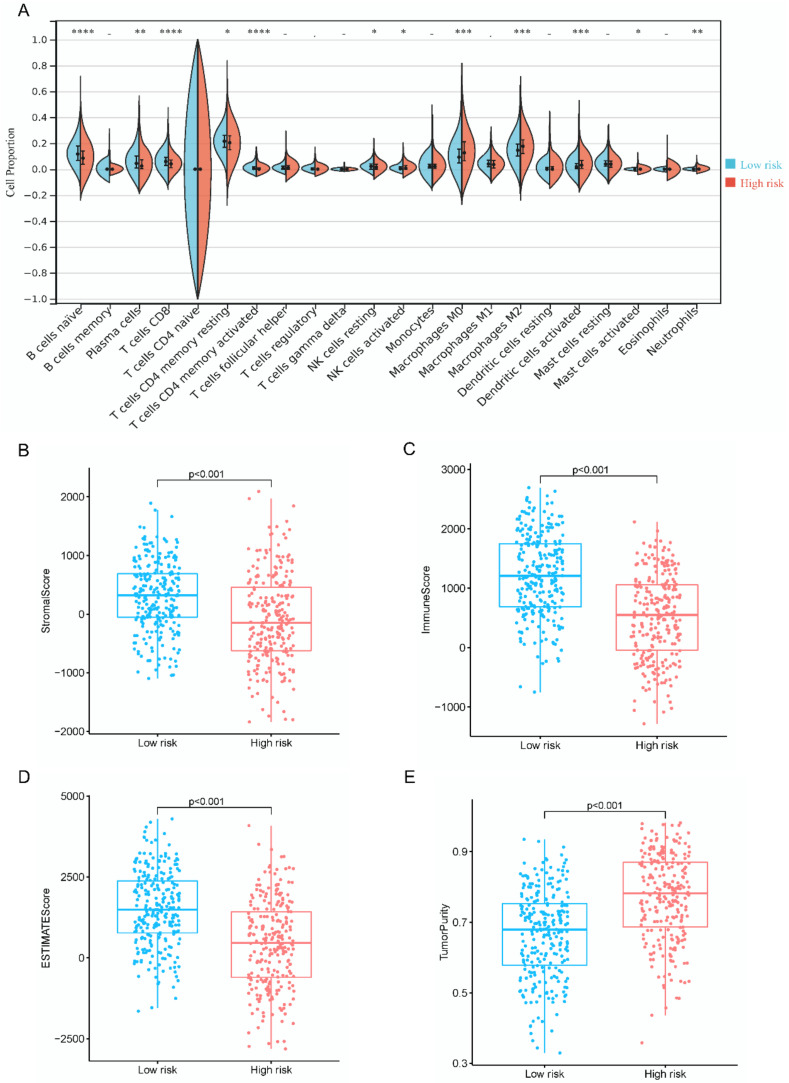
Immune microenvironment in the whole TCGA–LUAD set. Comparison of (**A**) 22 immune cell proportion, (**B**) stromal score, (**C**) immune score, (**D**) ESTIMATE score, and (**E**) tumor purity. Note: **** means *p* < 0.0001. *** means *p* <0.001. ** means *p* < 0.01. * means *p* < 0.05. - means no significant.

In order to characterize the immune environment of patients with LUAD, the proportions of infiltrating immune cells were compared between low- and high-risk groups in all samples using the CIBERSORT and LM22 signature matrices. The low-risk group had higher percentages of naive B cells, plasma cells, CD8+ T cells, and activated memory CD4+ T cells but lower percentages of M0 macrophages, M2 macrophages, and activated dendritic cells than the high-risk group (Figure 5A). Additionally, the low-risk group was found to have higher stromal scores (*p* < 0.001), immune scores (*p* < 0.001), and ESTIMATE scores (*p* < 0.001), but lower tumor purity (*p* < 0.001) than the high-risk group (Figure 5B–E).

**Figure 6:**
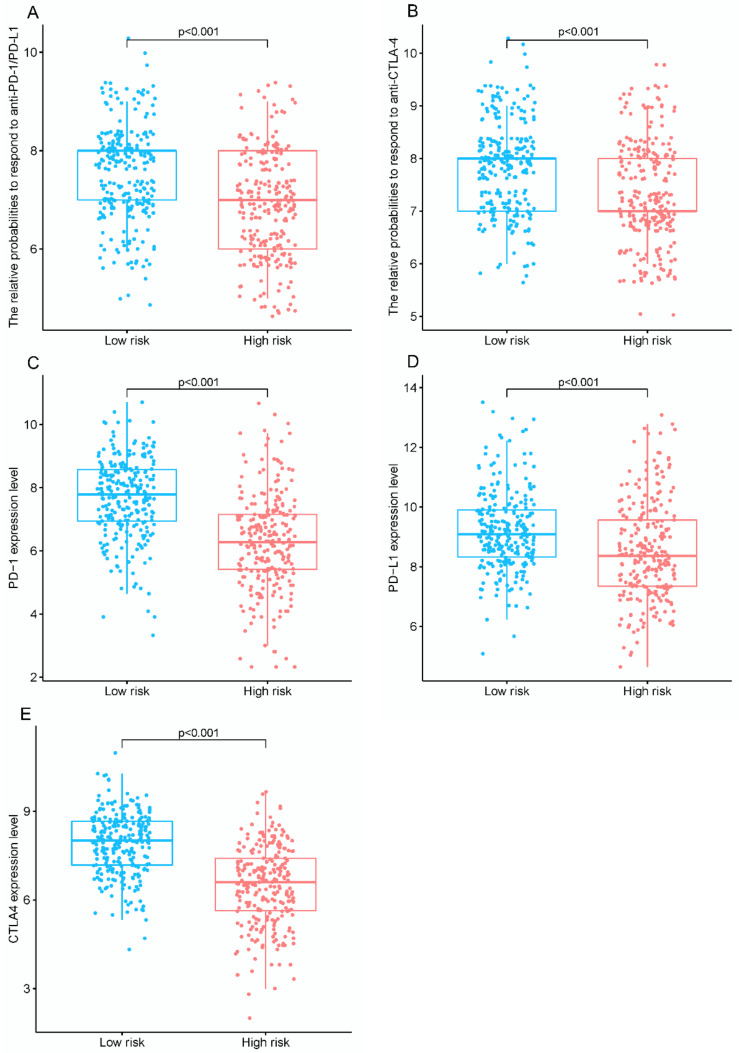
Predictive response to immune checkpoint inhibitor (ICI) treatment in patients with LUAD. Relative probabilities of responding to (**A**) anti-PD-1/PD-L1 and (**B**) anti-CTLA4 treatments. The mRNA levels of (**C**) PD-1, (**D**) PD-L1, and (**E**) CTLA4.

To explore the ability of the IRL signature in the prediction of immunotherapeutic sensitivity for LUAD patients, IPS values, which were calculated based on immunogenicity from the TCIA database, were analyzed in the risk model. The outcome showed that the potentials of the low-risk group to respond to anti-PD-1/PD-L1 (*p* < 0.001) and anti-cytotoxic T lymphocyte-associated antigen-4 (CTLA4, *p* < 0.001) treatment were higher than those of the high-risk group (Figure 6A,B). In accordance with these results, we found that the expression levels of PD-1 (*p* < 0.001), PD-L1 (*p* < 0.001), and CTLA4 (*p* < 0.001) were relatively increased in the low-risk group (Figure 6C–E). Thus, ICI treatment might be effective for patients with LUAD with low-risk scores. However, the expression profiles of the amplification of murine double minute (MDM) 2 and 4 (*p* = 0.002, *p* < 0.001), which were markers for HPD, were modestly elevated in low-risk patients (Figure S5), although no difference in DNA methyltransferase 3 alpha (DNMT3A), Cyclin D1, or Fibroblast Growth Factor (FGF) 3/4/19 was observed.

After conducting gene set enrichment analysis (GSEA) on the mRNAs of all LUAD samples, we found that more pathways in the low-risk group were upregulated compared with those in the high-risk group (Figure S6). Compared with the high-risk group, the low-risk group had significantly enriched V-Ki-Ras2 Kirsten rat sarcoma viral oncogene homolog (KRAS) signaling, interferon-gamma response, interleukin (IL) 2–signal transduction, and activator of transcription (STAT) 5 signaling. Moreover, class II major histocompatibility complex transactivator (CIITA) and interferon alpha and beta receptor subunit 2, as target mRNAs of AC104971.3 and LY86-AS1, took part in the interferon-gamma response, implying that IRLs might regulate the relevant pathways via target mRNAs and play an essential role in the pathogenesis of LUAD.

## 4. Discussion

Infiltrating immune cells in the tumor microenvironment are critical in cancer progression [25], and the quantitative evaluation of tumor immune infiltrates is still a major challenge using the traditional immunohistochemistry immunoscoring approach. Additionally, ICI has become the first-line treatment for advanced LUAD that is refractory to targeted therapy [5]. Nowadays. PD-L1 expression, tumor somatic mutation burden, mismatch repair deficiency, and microsatellite instability have been widely applied to predict ICI efficacy. However, several concerns, including the poor uniformity of detection technologies and different cutoff values for positivity across clinical trials, have limited its utility [26]. In recent years, IRLs have been proven to be indispensable in tumor progression and oncogenic pathways by regulating gene expression and can be used as a potential prognostic biomarker for cancer [27].

Immunologic features, which actively participate in cancer development, can more effectively predict patients’ survival than traditional intrinsic features of tumors [28]. With the dramatic development of gene sequencing technology, molecular profiling-based signatures to infer immune infiltration have become a reality. Several previous studies constructed risk models with novel IRLs that can predict the prognosis in patients with LUAD. In most of these studies, IRLs are obtained through different expressions combined with Pearson correlation or interaction prediction with immune-related mRNA expression profiles from databases. Another study selected lncRNAs that are upregulated in immune cell lines but downregulated in NSCLC cell lines as tumor-infiltrating IRLs [29]. LncRNAs are suggested to participate in the immune response by regulating the expression of target mRNAs and interacting with chromatin, proteins, and miRNA in various ways [30,31]. Hence, only a few IRLs have been found to play a role in LUAD so far, and high-throughput methods for the identification of lncRNAs affecting immune activity are still largely unknown.

Therefore, our study of several novel IRLs is an essential complement to identifying their roles in immune regulation and immunotherapy targets in LUAD. Here, we assessed lncRNAs directly related to immune response, which are systematically identified via a computational algorithm and represent immune pathways and distinct immune cell types in the immLnc database. Afterward, we extracted IRLs and relevant clinical information from TCGA–LUAD to construct a risk signature via integrative analysis and then divided patients with LUAD into low- and high-risk groups. Comparing the survival outcomes of patients classified by the six IRLs, we supposed that the risk score model and integrated nomogram have a reliable and stable prediction performance. Moreover, cytotoxic T cells, Th1 helper cells, B cells, and plasma cells eliminate tumor cells in the antitumor immune milieu, whereas specific macrophages and regulatory T cells can accelerate immune escape and tumor growth in the protumorigenic immune milieu [32]. Protective CD8+ T cell responses can be induced by activated dendritic cells in the inflammation of normal tissue [33], whereas an immune reaction to lung cancer in the presence of mature dendritic cells (activated or not) is necessary to organize cytotoxic T cells, which are associated with a good clinical outcome and response to therapeutics [34]. Similarly, we found that patients with low-risk immune signatures have long OS; increased B cells, plasma cells, CD8+ T cells, and CD4+ T cells; and minimal macrophages and activated dendritic cells infiltrated in tumors.

Some similar published papers studied IRLs by performing coexpression analysis between lncRNAs from TCGA and immune-related genes from MsigDB, ImmPort, or the GSEA database, of which only three papers were validated by GEO microarray data [22,23] or clinical studies [21]. We retrieved IRLs for LUAD from the ImmLnc database, which was established by integrating tumor purity estimation, GSEA, and powerful algorithms [9], and uninvestigated IRLs were found by directly retrieving IRLs from ImmLnc and TCGA and integrating bioinformatics. The risk model and nomogram presented here were validated by GEO sequencing data and our clinical study, suggesting that the IRL signature is available in Chinese patients with LUAD.

An effective IRL-based model for patient selection before ICI treatment in LUAD has not been studied yet. A considerable proportion of patients with NSCLC have a poor response to immunotherapy despite the high expression of immune checkpoint molecules [35]. Thus, developing comprehensive predictive biomarkers is indispensable. The complex interaction between tumor immune infiltrates and the immunotherapy response affects NSCLC [36]. For instance, more CD8+ T cell infiltration in lung cancer tissue is associated with a superior treatment response from pembrolizumab treatment [37]. In our model, patients in the low-risk group have increased IPS values for PD1/PD-L1 and CTLA4 blockers, and the expression of immune checkpoint molecules may be associated with improved sensitivity to ICI treatment, suggesting that their tumors are in a preactivated immune status. Thus, six IRLs can be useful for choosing suitable immunotherapy. Additionally, HPD is a novel pattern of tumor progression, with unexpected and rapid tumor growth, poor prognosis of patients, and high rates of fatality, which have limited the clinical application of ICI [6]. Considering the limitations of ICI due to HPD incidence, valid biomarkers are urgently needed to predict the occurrence of HPD to improve ICI efficacy. To date, several tumor cell biomarkers, including MDM2/4, epidermal growth factor receptor mutation, DNMT3A, and FGF3/4/19, have been shown to be associated with HPD [38]. We found that the expression profiles of MDM2 and MDM4, which regulate p53 and apoptotic responses to cellular injuries when overexpressed [39], are modestly elevated in low-risk patients. This finding implies that patients with a minimal IRL signature may gain benefits from ICI treatment, but some patients may have a potential risk of HPD. Hence, multiple factors influencing the efficacy of ICI should be comprehensively considered to optimize treatment regimens.

To explore the possible underlying mechanisms, we conducted GSEA on targeted mRNAs of IRLs. KRAS signaling, interferon-gamma response, and IL2–STAT5 signaling, as potential positive predictors of antitumor immunity [40], are enriched in the low-risk group. CIITA, the target mRNA of AC104971, plays an important role in the interferon-gamma response. Consistent with our result, the loss of CIITA converts lung cancer from anti-PD-1-sensitive to anti-PD-1-resistant [41]. Our study suggests that these IRLs may regulate target mRNAs and play a functional role in the sensitivity to ICI treatment for LUAD.

Even with the above promising findings, some limitations remain in this study. First, the prognostic model is established by public databases, which may increase the bias. Even though the model has been validated with the internal database, external database, and single-center case-control study, the optimal cut-off value and predictive capability of the six IRLs demand further confirmation in prospective clinical trials with complete survival time based on a large sample size. Second, the functions of the six IRLs have not been validated. Thus, functional and mechanistic experiments are needed to support our findings.

In conclusion, the six-IRL signature is a promising biomarker for prognosis prediction and facilitates the management of immunotherapy in LUAD. Patients with low risk might gain benefits from ICI, although some have a risk of hyperprogressive disease.

## Figures and Tables

**Table 1:** Severity of patients with lung adenocarcinoma (LUAD) and a low-risk score vs. patients with LUAD and a high-risk score in the case-control study.

	Low Risk	High Risk	*p* Value
TNM			
Stage I + II	9	1	0.005 **
Stage III + IV	6	14	
T stage			
1–2	9	4	0.139
3–4	6	11	
M stage			
0	11	2	0.003 **
1	4	13	
N stage			
0	9	2	0.021 *
1–3	6	13	
Performance Status			
0	13	6	0.021 *
1–4	2	9	

Note: ** *p* < 0.01 is considered statistically significant. * *p* < 0.05 is considered statistically significant.

## Data Availability

The data that support the findings of this study are openly available in the TCGA cohort of the UCSC Xena database (https://xenabrowser.net/ accessed on 16 September 2020), the GEO database (https://www.ncbi.nlm.nih.gov/geo/ accessed on 4 July 2022), and the immLnc database (http://bio-bigdata.hrbmu.edu.cn/ImmLnc/ accessed on 28 March 2021). The version 22 references that genome data, IPS data, and target mRNAs of IRLs were obtained from the Gencode database (https://www.gencodegenes.org/ accessed on 27 April 2020), The Cancer Immunome Atlas database (https://tcia.at/home accessed on 10 May 202), and the starbase3.0 database (http://starbase.sysu.edu.cn/ accessed on 6 April 2020).

## Supplementary Materials

The following supporting information can be downloaded at: https://www.mdpi.com/article/10.3390/diagnostics12112891/s1, Table S1: Clinicopathological characteristics of LUAD patients in TCGA, GEO datasets and clinical cohort; Table S2: Baseline clinical characteristics of LUAD patients with low and high risk in TCGA, GEO datasets, and clinical cohorts; Table S3: Primer sequence of lncRNAs; Figure S1: Expression profiles of the prognostic immune signature; Figure S2: ROC curves of risk score with IRLs; Figure S3: Absolute quantification of six IRLs in a case-control study; Figure S4: Comparison with other reported IRL risk score models and relevant nomograms; Figure S5: Biomarkers for hyperprogression in response to ICI treatment; Figure S6: Top 10 hallmark pathways of GSEA for patients with LUAD and low risk score vs. patients with LUAD and high risk score.
