# Supplementary material for: Identification and Application of a Novel Immune-Related lncRNA Signature on the Prognosis and Immunotherapy for Lung Adenocarcinoma
Source: Diagnostics (Basel). 2022 Nov 21;12(11):2891. doi: 10.3390/diagnostics12112891 (PMC9689875; doi:10.3390/diagnostics12112891)
Supplement: Supplementary file 1 [file diagnostics-12-02891-s001.zip › diagnostics-1954021-supplementary.pdf]

Descriptions of supplement

Table S1. Clinicopathological characteristics of LUAD patients in TCGA, GEO datasets and clinical cohort.

	TCGA dataset			GEO dataset	Clinical cohort
	Training	Testing	Entire	GSE120622	
	(N=360)	(N=153)	(N=513)	(N=44)	(N=30)
Gender					
Male	167 (46.4%)	70 (45.8%)	237 (46.2%)	24 (54.5%)	18 (60.0%)
Female	193 (53.6%)	83 (54.2%)	276 (53.8%)	20 (45.5%)	12 (40.0%)
Age (years)					
>60	244 (67.8%)	96 (62.7%)	340 (66.3%)	30 (68.2%)	22 (73.3%)
≤60	111 (30.8%)	52 (34.0%)	163 (31.8%)	14 (31.8%)	8 (26.7%)
Unknown	5 (1.4%)	5 (3.3%)	10 (1.9%)	NA	NA
TNM stage					
Stage I	199 (55.3%)	81 (52.9%)	280 (54.6%)	18 (40.9%)	8 (26.7%)
Stage II	83 (23.1%)	37 (24.2%)	120 (23.4%)	8 (18.2%)	2 (6.7%)
Stage III	56 (15.6%)	24 (15.7%)	80 (15.6%)	17 (38.6%)	3 (10.0%)
Stage IV	18 (5.0%)	7 (4.6%)	25 (4.9%)	1 (2.3%)	17 (56.7%)
Unknown	4 (1.1%)	4 (2.6%)	8 (1.6%)	NA	NA
T stage					
T1	120 (33.3%)	51 (33.3%)	171 (33.3%)	4 (9.1%)	5 (16.7%)
T2	191 (53.1%)	84 (54.9%)	275 (53.6%)	31 (70.5%)	8 (26.7%)
T3	31 (8.6%)	15 (9.8%)	46 (9.0%)	6 (13.6%)	5 (16.7%)
T4	15 (4.2%)	3 (2.0%)	18 (3.5%)	2 (4.5%)	12 (40.0%)
Unknown	3 (0.8%)	NA	3 (0.6%)	1 (2.3%)	NA
M stage					
M0	240 (66.7%)	102 (66.7%)	342 (66.7%)	42 (95.5%)	13 (43.3%)
M1	17 (4.7%)	7 (4.6%)	24 (4.7%)	1 (2.3%)	17 (56.7%)
MX	101 (28.1%)	41 (26.8%)	142 (27.7%)	NA	NA
Unknown	2 (0.6%)	3 (2.0%)	5 (1.0%)	1 (2.3%)	NA
N stage					
N0	240 (66.7%)	95 (62.1%)	335 (65.3%)	23 (52.3%)	11 (36.7%)
N1	62 (17.2%)	32 (20.9%)	94 (18.3%)	10 (22.7%)	NA
N2	47 (13.1%)	22 (14.4%)	69 (13.5%)	11 (25.0%)	13 (43.3%)
N3	1 (0.3%)	1 (0.7%)	2 (0.4%)	NA	6 (20.0%)
NX	9 (2.5%)	3 (2.0%)	12 (2.3%)	NA	NA
Unknown	1 (0.3%)	NA	1 (0.2%)	NA	NA

Note: N means Number; NA means Not available.

Table S2. Baseline clinical characteristics of LUAD patients with low and high risk in TCGA, GEO datasets and clinical cohort.

		Low risk (N)	High risk (N)	p value
TCGA dataset				
	Gender			0.1467
	Male	111	126	
	Female	147	129	
	Age (years)			0.0761
	>60	181	159	
	≤60	73	90	
	Unknown	4	6	
GSE 120622				
	Gender			0.2259
	Male	14	10	
	Female	8	12	
	Age (years)			>0.9999
	>60	15	15	
	⩽60	7	7	
Clinical cohort				
	Gender			0.7104
	Male	8	10	
	Female	7	5	
	Age (years)			0.2148
	>60	13	9	
	≤60	2	6	

Note: N means Number.

Table S3. Primer sequence of lncRNAs.

Primer name	Primer sequence	Length
AC104971.3	TCTTGGCACCTAACAGTTTGTGAA<br>GTTCTCAGTCCTCTGGGATTTCT	70bp
FAM215A	CACCGTTTGGGATGGTTGATT<br>GCTCCTTATTTAACGCACTGTTG	139bp
AC021678.2	GGACTAACCCAGGAAGATGCTAC<br>CCAGCTGATACCCTTGGACTCT	81bp
LINC02413	CGTAGCAACTGATTGGATTTCTG<br>TCTTAGCGTAACATCCATCATGC	122bp
AL161781.2	GTAGACTCTTTGGACAGTATGGGA<br>A CTCAAAACAAACAACCACCACA	178bp
LY86-AS1	AAGGCATACTGAGTTGCGGG<br>TCTGTTCGGTTCAAGAAAGGG	74bp
GADPH	ACCCAGAAGACTGTGGATGG<br>TTCTAGACGGCAGGTCAGGT	20bp

Supplementary figures with legends

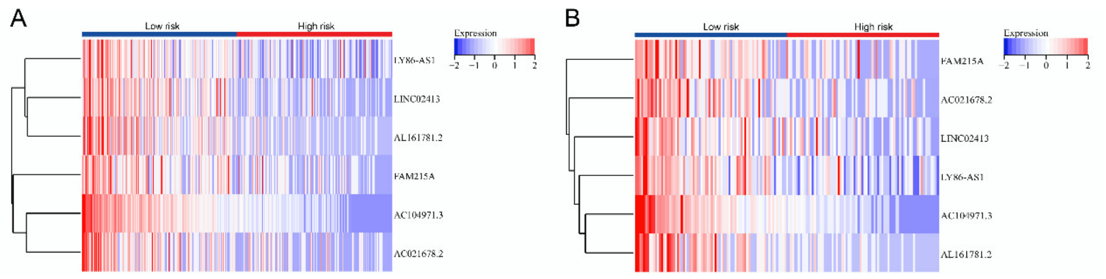

Figure S1. Expression profiles of the prognostic immune signature. Heatmap of expression in the six prognostic IRLs from (A) training and (B) testing TCGA datasets.

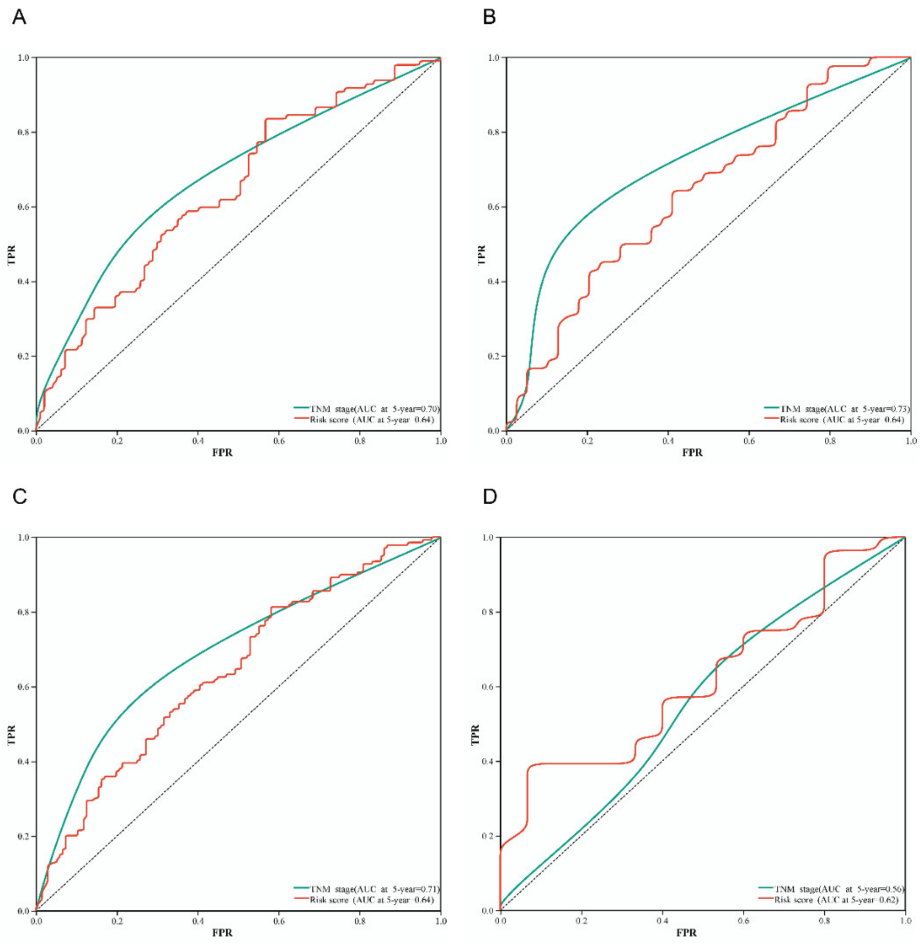

Figure S2. ROC curves of risk score with IRLs. ROC curves for predicting 5-year overall survival in patients of the (A) TCGA training dataset, (B) TCGA testing dataset (C) TCGA entire dataset, and (D) GSE120622. AUC, areas under the ROC curve. FPR, false positive rate. TPR, true positive rate.

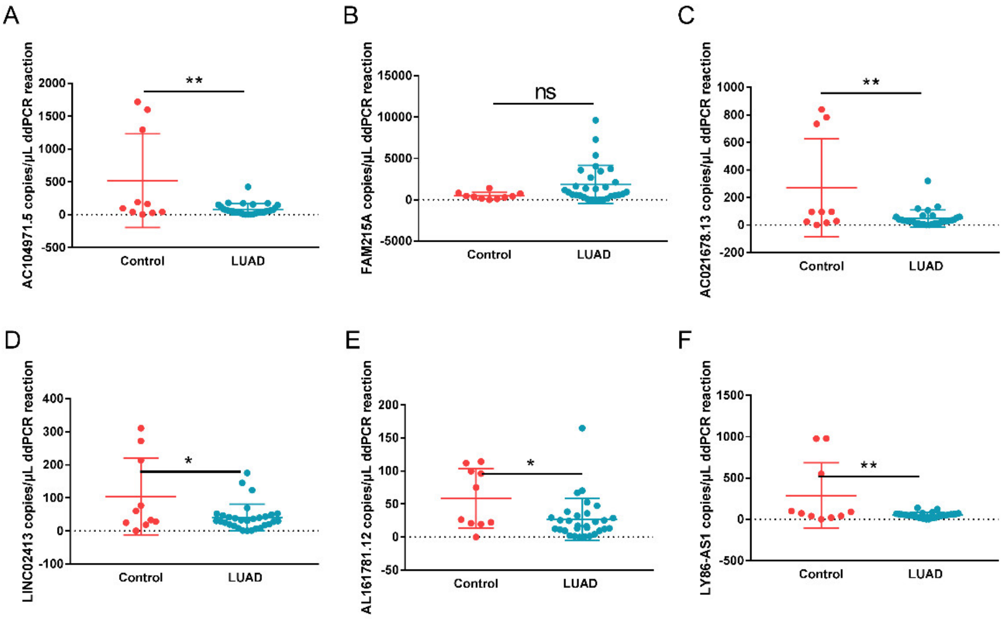

Figure S3. Absolute quantification of six IRLs in a case-control study. (A–F) Expression profiles of AC104971.5, FAM215A, AC021678.13, LINC02413, AL161781.12, and LY86-AS1 in lung tissue from 30 patients with LUAD and 10 negative controls. **$p < 0.01$, *$p < 0.05$.

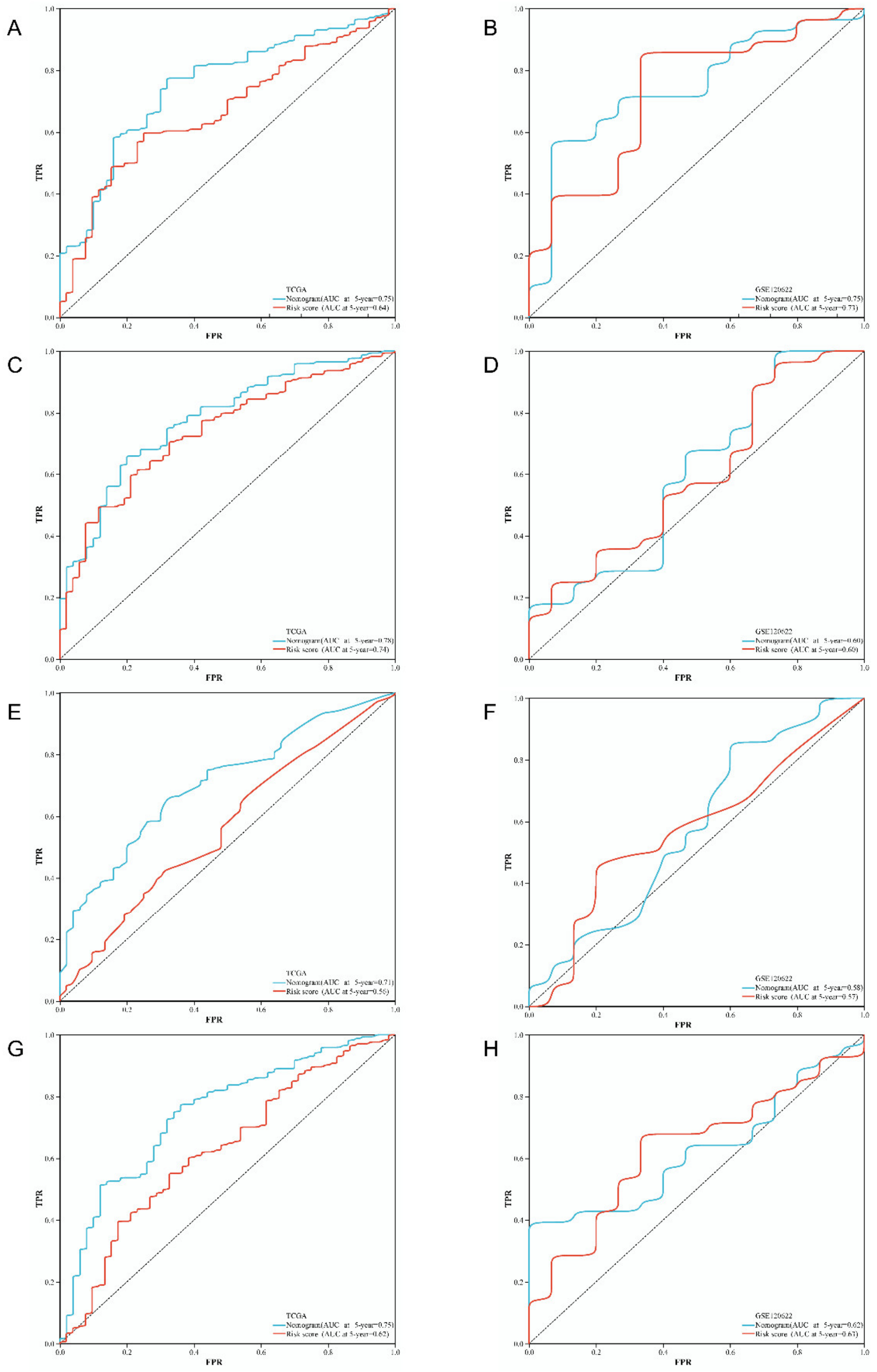

Figure S4. Comparison with other reported IRL risk score models and relevant nomograms. AUC values of the ROC curves of (A, B) 4-IRL signature, (C, D) 33 IRL pairs signature, (E, F) 8 lRL pairs signature, and (G, H) 6-IRL signature in TCGA–LUAD entire dataset and GSE120622.

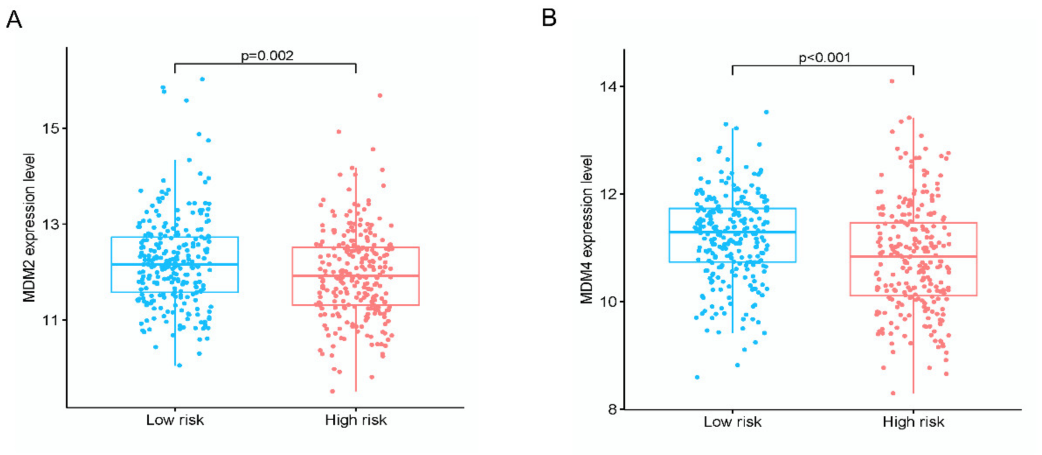

Figure S5. Biomarkers for hyperprogression in response to ICI treatment. mRNA levels of (A) MDM2 and (B) MDM4 in low- and high-risk groups.

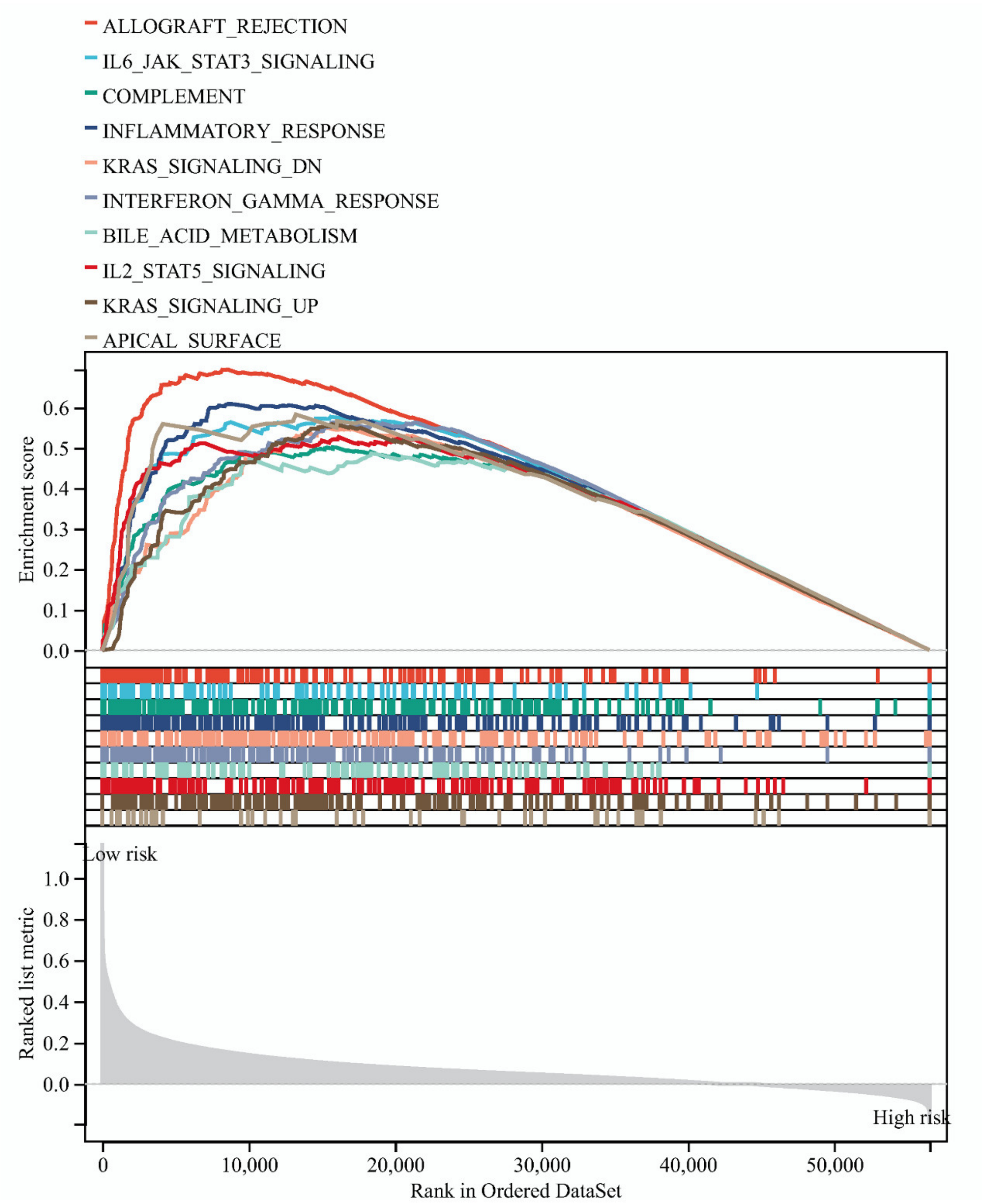

Figure S6. Top 10 hallmark pathways of GSEA for patients with LUAD and low risk score vs. patients with LUAD and high risk score.
